# Proteases: Essential Actors in Processing Antigens and Intracellular Toll-Like Receptors

**DOI:** 10.3389/fimmu.2013.00299

**Published:** 2013-09-24

**Authors:** Bénédicte Manoury

**Affiliations:** ^1^Institut National de la Santé et de le Recherche Médicale, Unité 1013, Université Paris Descartes, Sorbonne Paris Cité, Faculté de médecine, Paris, France

**Keywords:** antigen processing, MHC class II, endosomal proteases, intracellular toll-like receptors

## Abstract

MHC class II molecules expressed by professional antigen presenting cells (pAPCs) such as macrophages, B cells, and dendritic cells (DCs) play a fundamental role in presenting peptides to CD4^+^ T cells. However, to elicit CD4^+^-T cells immunity, pAPCs need an additional signal, which can be delivered by toll-like receptors (TLRs) molecules. TLRs recognize microbial patterns and are critical in initiating immune responses. Proteases, which provide peptide ligands for the MHC class II antigenic presentation pathway, were recently shown to cleave and activate intracellular TLRs in endosomal compartments. Here, I give an overview on the individual roles of the most well studied proteases in both antigen and TLRs processing.

## MHC Class II Pathway

A complex series of biosynthetic and proteolytic events must ensure correct assembly, trafficking, and peptide loading in order for the MHC class II molecules to efficiently interact with their specific T-cell Receptor (TCR) expressed on CD4^+^ helper T cells (1, 2). MHC class II molecules consist of an α and β chain which dimerize shortly after synthesis in the endoplasmic reticulum (ER) to create the groove in which antigenic peptides are accommodated. Newly synthesized αβ dimers assemble with a non-polymorphic glycoprotein, the invariant chain (Ii), which fills the peptide-binding groove of αβ dimers and prevent loading of premature peptides in the ER. In addition, the cytoplasmic tail of Ii contains targeting signals that deliver the MHC class II-Ii complexes from the trans-Golgi network directly into the endocytic pathway (3) where exogenous antigen are internalized. Ii is then sequentially cleaved leaving a C-terminal (C-ter) portion: the class II-associated invariant chain peptide or CLIP in the peptide-binding groove. Within the lysosomes, the chaperone molecule HLA-DM interacts with MHCII-CLIP and catalyzes the exchange of CLIP for exogenous peptides. Newly formed MHCII-peptides complexes are then targeted to the plasma membrane where they interact with CD4^+^ T cells.

## Antigen Processing

The presentation of antigens by MHC class II molecules is strictly dependent on the action of endocytic proteases. Indeed, these enzymes not only degrade proteins in order to produce the antigenic peptides but they also process the Ii chain. Limited antigen proteolysis is required for MHC class II-peptide loading and peptides of 9–16 residues are presented to CD4^+^ T cells. A balanced proteolytic environment is therefore required to ensure adequate antigen processing while preventing complete destruction. The three main classes of intracellular proteases residing in the lysosomal/endosomal compartments and participating in antigen degradation are cysteine (cathepsin B, F, H, L, S, Z, and AEP, for asparaginylendopeptidase), aspartate (cathepsin D, E), and serine (cathepsin A, G) proteases. The protease nomenclature designates the amino acid of the protease active site that catalyzes hydrolysis of the substrate peptide bond. With the exception of AEP, most endocytic proteases display broad cleavage specificity. Indeed, AEP cleaves on the carboxyl terminal sides of asparagines residues whereas other cysteine proteases recognize hydrophobic motifs.

The use of specific protease inhibitors and mice deficient for murine proteases has helped to identify the key enzymes in Ii processing. Indeed, the final steps of Ii degradation are dependent on cathepsin L or S and in their absence an Ii N-terminal fragment of approximately of 10 kDa (p10) accumulates on MHC class II molecules (4–6). However, it is less obvious which enzymes are involved in antigen processing but a critical role of cathepsin S and L has been confirmed in the generation of MHC II-peptides complexes albeit in different cell types (4–8). In addition, cathepsin F was reported to compensate for the loss of cathepsins S and L in macrophages (9). Furthermore, in a recent human study, cathepsin S expressed in thymic dendritic cells (DCs) was shown to be responsible for the destruction of a certain number of epitopes from two auto-antigens involved in experimental allergic encephalitis and in diabetes (10).

Concerning serine proteases, a latest study has described the importance of cathepsin G in generating several proinsulin peptides *in vitro* and in human cells. Indeed, cathepsin G activity was found to be elevated in PBMC from diabetic patients and blocking its activity resulted in the abrogation of the proliferation of specific proinsulin T cells (11).

Together the groups of Alan Barrett and Colin Watts described few years ago a novel lysosomal cysteine protease, AEP. This asparagine endopeptidase was shown to initiate processing of the tetanus toxin antigen in human B cells (12, 13), to be capable of destroying an immunodominant peptide of myelin basic protein (MBP, 85–99) an auto antigen implicated in the autoimmune disease multiple sclerosis (14) and to perform the early step of Ii chain degradation in human B-EBV cells (15). AEP participation was clearly demonstrated in processing antigens in human APCs. However, its role was less clear in mice. Indeed, it was reported that in AEP-deficient cells, antigen processing of Ii chain and two other antigens were weakly altered (16, 17).

An additional lysosomal processing enzyme, named GILT or gamma interferon inducible lysosomal thiol reductase, was described to generate T cell epitopes by reducing proteins disulfide bonds. Indeed, by catalyzing the reduction of disulfide bonds, GILT generates proteins partially denatured more susceptible to proteolysis. GILT is now reported to be required for the presentation of many peptide ligands of hen egg lysozyme, ribonuclease A, human IgG, the melanoma differentiation antigens tyrosine and TRP1, the human immunodeficiency virus-1 envelope proteins and the allergens Derp1 and Blag2 which all contain disulfide bonds (18–22).

Another important role of AEP and GILT is to regulate the expression or the maturation of other cysteine proteases. Lysosomal proteases reach the endocytic compartments as proforms or zymogens where their propeptide is removed by proteolysis. The resulting single-chain form is then cleaved into a mature form or a two-chain form (heavy and light chains). For example, GILT has been described to regulate the expression of cathepsin B specifically in B cells (23) and AEP-deficient mice exhibit a defect in the maturation of Cat B, Cat D, Cat H, and Cat L in kidney and bone marrow derived dendritic cells (BMDCs) (16, 24, 25) and an increase in Cat K expression. How exactly AEP mediates these events are still unclear.

Acidic pH is a prerequisite for maturation and activity of most of these intracellular proteases and so their maximal activity is found in lysosomal compartments. Indeed, chloroquine, a lysomotropic agent, which was shown to abrogate MHC class II antigen presentation because of inhibiting enzymatic activities in lysosomes, also altered intracellular toll-like receptors (TLRs) signaling (26). Accordingly, a new role for the endocytic proteases was discovered.

## TLRs Processing

Toll-like receptors are proteins, which recognize conserved molecules from microorganisms and in DCs, they are crucial in linking innate to adaptive immunity. TLRs contain several leucine rich repeats (LRR) in an extracellular loop, a trans-membrane domain, and a cytosolic domain and are expressed either at the plasma membrane or in the endosomal/lysosomal organelles. TLR stimulation is linked to MyD88 or TRIF-dependent signaling pathways that regulate the activation of different transcription factors, such as NF-κB and IRF (27). Specific interaction between TLRs and their ligands activates NF-κB resulting in enhanced inflammatory cytokine responses, induction of DCs maturation and expression of chemokine receptors. TLRs expressed at the plasma membrane recognized Gram-negative bacteria and endosomal TLRs sense viral and bacterial nucleic acids such as double/single-stranded RNA or double stranded DNA. Endogenous ligands called DAMPs (for damage associated molecular patterns) may also activate TLRs during self-tissues or cell damage (28). Several published results demonstrated that intracellular TLRs require partial proteolysis in endosomes for full-activation. Indeed, many groups have now reported that murine TLR9 is non-functional until it is subjected to proteolytic cleavage in the endosomes (24, 29–31). Upon stimulation, full-length (FL) TLR9 is cleaved into a C-ter fragment sufficient for signaling. This cleavage is realized by several proteases in different cells. Addition of Z-FA-FMK, a broad inhibitor of cathepsins, or specific inhibitors of cathepsins B, L, and S partially or completely impaired TLR9 signaling in macrophages and B cell lines. In primary cells such as BMDCs, deficiency of cathepsin L led to partial reduction (about 50%) of TLR9 function. In cathepsin K deficient DCs, TLR9 signaling was totally abrogated (32). Moreover, inhibition of cathepsin K activity exerted beneficial effects on collagen-induced arthritis in mice, an autoimmune disease induced after injection of type II collagen and complete freund adjuvant containing TLR9 agonist such as CpG-ODN (32). In addition, in DCs and mice lacking AEP, even though TLR9 cleavage in phagosomal compartments was still occurring, CD4^+^ antigen specific T cell proliferation was greatly reduced upon CpG and ovalbumin stimulation (24). These results correlated with some *in vitro* digestion assays where FL TLR9 was shown to be proteolyzed into a C-ter fragment by cathepsins K, L, S, or AEP (24, 29–31). Altogether, these results described clearly the involvement of several distinct lysosomal proteases for TLR9 function.

Concerning TLR7, the literature is scarce but it was firstly reported the generation of a TLR7 proteolytic fragment (33) on a SDS gel, which by analogy with TLR9 was identified as the TLR7 C-ter fragment. Then, using different wild type or AEP-deficient primary cells, such as DCs, plasmacytoid dendritic cells (pDCs) or epithelial cells, it was shown that TLR7 was also subjected to similar proteolytic maturation than TLR9 and required AEP for proper signaling. In addition, infected mice lacking AEP with influenza virus, a single-stranded RNA sensed by TLR7, developed much less inflammation and exhibited significant reduced CD8^+^ T cells priming (34). Recently, it was shown that, in contrast to TLR9, TLR7 N-terminal fragment (N-Ter) remained linked by a disulfide bond with TLR7 C-ter. Cysteine 98 in TLR7 N-ter and Cysteine 445 in TLR7 C-ter were required for this disulfide bond and mutating one of them abrogated TLR proteolytic cleavage and RNA sensing (35). It will be interesting to investigate whether this disulfide bond in TLR7 is also required for TLR7 signaling in pAPCs such as DCs and the eventual role of GILT in maintaining or reducing this disulfide bond. Nevertheless, it still uncertain whether or not human TLR7 and TLR9 require proteolysis for their function. Only one report to date has shown that blocking AEP activity in human pDCs totally abrogate TLR9 signaling (24).

The results concerning TLR3 processing are less obvious. In the mouse and human system, it was described that Z-FA-FMK did alter TLR3 processing but not its signaling in a macrophage cell line and in HEK 293 cells overexpressing TLR3 and UNC93B1 (36). In contrast, inhibition of TLR3 processing into a C-ter fragment and subsequently its signaling was reported by the group of S. Lebecque in human monocyte derived DCs incubated with Z-FA-FMK (37). Moreover, the identity of the proteases involved in TLR3 proteolysis is still a matter of debate. Yet, cathepsins B and H seem to be good candidates (38) to generate the TLR3 C-terminal fragment as it was observed that in cells silenced for these two genes, the generation of the TLR3 C-ter fragment was reduced.

Intracellular TLRs traffic from the ER to lysosomal compartments where they respond to their ligands. UNC93B1, an ER resident protein, facilitates their trafficking (39, 40). In mice and cells defective for UNC93B1, harboring a mutation in the trans-membrane domain, TLR7 and TLR9 remain in the ER and fail to respond to TLRs stimulation. As a consequence, mice expressing mutated UNC93B1 (3d mice) are more susceptible to bacterial and viral infection (41, 42). Beyond the role of UNC93B1 in intracellular TLRs trafficking, UNC93B1 is also important in the MHC class I cross presentation and MHC class II pathways (42). Indeed, mice and DCs mutated for UNC93B1 are unable to present exogenous antigens to CD4^+^ and CD8^+^ T cells. However, despite abundant work on UNC93B1, little is known about the molecular mechanism leading to MHC II antigen presentation defect in pAPCs expressing a faulty UNC93B1 protein. Interestingly, recently, MHCII has been shown to promote full-activation of TLR9. Following stimulation of TLR3 and 9, MHCII forms a complex with CD40 and the phosphorylated Bruton tyrosine kinase (Btk) in lysosomes (43). This prolonged interaction maintains Btk activated and increases proinflammatory cytokines and type I interferon secretion by DCs and macrophages following TLRs stimulation.

In addition to the role of TLRs in DCs activation via the up regulation of costimulatory molecules, TLR7 and 9 stimulation induce a drop of pH in the early endosomes of DCs (24, 34). This acidic pH, which boost protease activities, probably also favor processing of exogenous antigen and MHC class II presentation.

## Conclusion

**Figure 1:**
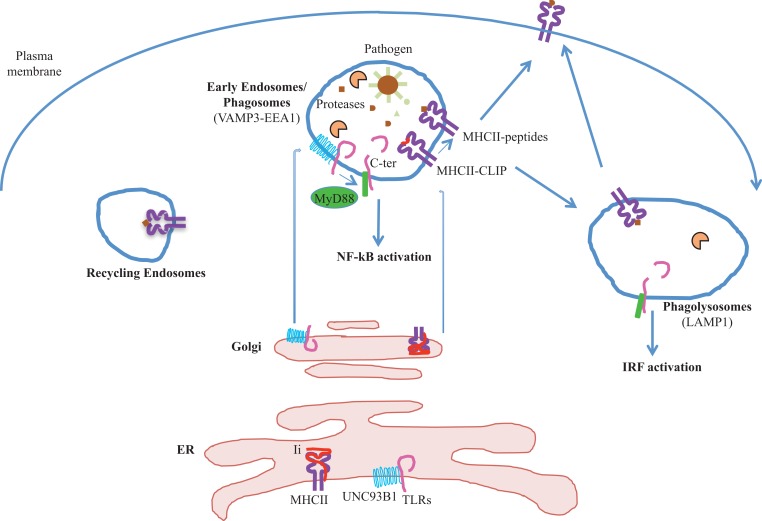
**Endosomal proteases process internalized antigens and intracellular TLRs**. Both intracellular TLRs and MHCII molecules associate with their specific chaperones proteins in the ER, UNC93B1, and Ii chains respectively, and traffic toward the endosomes. In the endosomes, TLRs, Ii chain, and exogenous antigens are cleaved by proteases. Peptides are loaded on MHCII molecules and the complexes MHCII-peptides are then target to the plasma membrane to interact with their specific TCR expressed by CD4^+^ T cells. Cleaved TLRs associate with the adaptor molecule MyD88 that trigger either NF-κB or IRF activation. These two events, formation of MHC II-peptide complexes and TLRs activation in APCs, are critical for inducing CD4 T cell responses.

The endosomal/phagosomal pathway is a key meeting point between proteins regulating innate and adaptive immunity (Figure 1). MHCII, UNC93B1, and proteases have been shown to regulate both TLRs signaling and MHCII presentation. Understanding how proteases are regulated in specific APCs and identifying new components in TLRs activation, especially upon pathogen infection, will be no doubt important for controlling specific unwanted immune responses.

## Conflict of Interest Statement

The authors declare that the research was conducted in the absence of any commercial or financial relationships that could be construed as a potential conflict of interest.
